# An Exercise Intervention to Unravel the Mechanisms Underlying Insulin Resistance in a Cohort of Black South African Women: Protocol for a Randomized Controlled Trial and Baseline Characteristics of Participants

**DOI:** 10.2196/resprot.9098

**Published:** 2018-04-18

**Authors:** Julia H Goedecke, Amy E Mendham, Louise Clamp, Pamela A Nono Nankam, Melony C Fortuin-de Smidt, Lindokuhle Phiri, Lisa K Micklesfield, Dheshnie Keswell, Nicholas J Woudberg, Sandrine Lecour, Ali Alhamud, Mamadou Kaba, Faith M Lutomia, Paul J van Jaarsveld, Anniza de Villiers, Steven E Kahn, Elin Chorell, Jon Hauksson, Tommy Olsson

**Affiliations:** ^1^ Non-Communicable Diseases Research Unit South African Medical Research Council Cape Town South Africa; ^2^ Division of Exercise Science and Sports Medicine Department of Human Biology University of Cape Town Cape Town South Africa; ^3^ South African Medical Research Council / University of the Witwatersrand Developmental Pathways for Health Research Unit Faculty of Health Sciences University of the Witwatersrand Gauteng South Africa; ^4^ Hatter Institute for Cardiovascular Research in Africa Department of Medicine University of Cape Town Cape Town South Africa; ^5^ Division of Biomedical Engineering Department of Human Biology University of Cape Town Cape Town South Africa; ^6^ Division of Medical Microbiology Department of Pathology University of Cape Town Cape Town South Africa; ^7^ Division of Medical Physiology Faculty of Medicine and Health Sciences Stellenbosch University Cape Town South Africa; ^8^ Division of Metabolism, Endocrinology and Nutrition Department of Medicine Veterans Affairs Puget Sound Health Care System and University of Washington Seattle, WA United States; ^9^ Department of Public Health and Clinical Medicine Umeå University Umea Sweden; ^10^ Department of Radiation Sciences Umeå University Umeå Sweden

**Keywords:** diabetes mellitus, type 2, insulin resistance, body fat distribution, adipose tissue, skeletal muscle, gastrointestinal microbiome, exercise, fatty liver, inflammation, energy metabolism, cardiorespiratory fitness, lipids, metabolomics, fatty acids, diet records, mitochondria, ectopic fat

## Abstract

**Background:**

The pathogenesis of type 2 diabetes (T2D) in black African women is complex and differs from that in their white counterparts. However, earlier studies have been cross-sectional and provide little insight into the causal pathways. Exercise training is consistently used as a model to examine the mechanisms underlying insulin resistance and risk for T2D.

**Objective:**

The objective of the study was to examine the mechanisms underlying the changes in insulin sensitivity and secretion in response to a 12-week exercise intervention in obese black South African (SA) women.

**Methods:**

A total of 45 obese (body mass index, BMI: 30-40 kg/m^2^) black SA women were randomized into a control (n=22) or experimental (exercise; n=23) group. The exercise group completed 12 weeks of supervised combined aerobic and resistance training (40-60 min, 4 days/week), while the control group maintained their typical physical activity patterns, and both groups were requested not to change their dietary patterns. Before and following the 12-week intervention period, insulin sensitivity and secretion (frequently sampled intravenous glucose tolerance test) and its primary and secondary determinants were measured. Dietary intake, sleep quality and quantity, physical activity, and sedentary behaviors were measured every 4 weeks.

**Results:**

The final sample included 20 exercise and 15 control participants. Baseline sociodemographics, cardiorespiratory fitness, anthropometry, cardiometabolic risk factors, physical activity, and diet did not differ between the groups (*P*>.05).

**Conclusions:**

The study describes a research protocol for an exercise intervention to understand the mechanisms underlying insulin sensitivity and secretion in obese black SA women and aims to identify causal pathways underlying the high prevalence of insulin resistance and risk for T2D in black SA women, targeting specific areas for therapeutic intervention.

**Trial Registration:**

Pan African Clinical Trial Registry PACTR201711002789113; http://www.pactr.org/ATMWeb/ appmanager/atm/atmregistry?_nfpb=true&_pageLabel=portals_app_atmregistry_portal_page_13 (Archived by WebCite at http://www.webcitation.org/6xLEFqKr0)

## Introduction

### Background

Type 2 diabetes (T2D) is a significant contributor to morbidity and mortality worldwide [1]. Globally, sub-Saharan Africa has the highest projected rate of increase in T2D over the next 25 years, increasing by 2.5-fold from 14.2 million in 2015 to 34.2 million by 2040 [1]. T2D and its associated morbidity and mortality rates are more prevalent in populations of black African origin than white populations [2-4]. Within South Africa (SA), the prevalence of T2D has increased significantly over the past 20 years, particularly in black African urban-dwelling populations [5]. Higher prevalence rates in SA are found in women (14.7%) compared with men (11.3%) [5]. This high T2D rate is compounded by the high prevalence of obesity and insulin resistance in black women [6-8]. Insulin resistance in black populations is associated with hyperinsulinemia, as a result of greater insulin secretion and reduced hepatic insulin clearance [7-9]. However, with increasing age, insulin secretion in relation to insulin sensitivity decreases in black women and is associated with impaired glucose tolerance and T2D [10].

The pathogenesis of insulin resistance in black women is complex and differs from that in their white counterparts [11]. Despite greater insulin resistance, black women have less visceral adipose tissue (VAT) but more peripheral (gluteal-femoral) subcutaneous adipose tissue (SAT) deposition [12-14]. This paradox may be explained, in part, by differences in adipose tissue biology [11]. Compared with white women, SAT of black women is hypertrophic, has a reduced adipogenic capacity [15], a higher inflammatory profile [16], less vascularization, and increased fibrosis [17]. These findings are suggestive of pathological adipose tissue expansion, which is typically associated with ectopic fat deposition and insulin resistance [18]. However, we found that obese black women accumulated less hepatic fat than their white counterparts [19], which corresponds with their lower VAT levels [12], but had similar intra- and intermyocellular lipid content of the soleus muscle [19]. Furthermore, the association between ectopic fat and insulin sensitivity was more robust in black as compared with white women [19]. These distinct obesity-related phenotypic differences may differentially impact the risk for insulin resistance and T2D in black and white women. However, these studies were cross-sectional and provide little insights into the causal pathways involved.

Exercise training, via its effects on multiple organs and systems, reduces insulin resistance and the risk of T2D (for reviews [20-22]). Recent studies have suggested an important crosstalk between skeletal muscle, liver, adipose tissue and the pancreas [23-25], which is altered in response to exercise training [23]. The effects of exercise training on insulin sensitivity are primarily through insulin action in skeletal muscle [26], which involves many mechanisms, including changes in the insulin signaling, inflammation, reactive oxygen species (ROS), metabolic flexibility, mitochondrial biogenesis, and ectopic fat accumulation. Within adipose tissue, exercise training decreases the obesity-induced dysregulated expression of adipokines, adipocyte size, ROS and inflammation, and increases vasculature [27]. In addition, the importance of the gastrointestinal microbiome for diabetes risk has recently been recognized [28] and is responsive to exercise [29,30]. Indeed, advances in omics technologies have improved our understanding of systems biology in diseased states and can be used to identify novel pathways underlying insulin resistance and T2D risk.

Although, we can identify biological and physiological correlates of insulin resistance in black women, these may merely reflect adaptations to environmental and lifestyle factors. There are marked ethnic differences in socioeconomic status, dietary intake, and physical activity between black and white SA women [12]. In terms of physical activity, black women accumulate activity through walking for transport (typically performed at a low intensity), while participation in leisure activities that are generally performed at moderate-to-high intensities is uncommon [12,31]. Accordingly, black women have very low cardiorespiratory fitness levels, which associates with their high levels of obesity and insulin resistance [32].

Overall, exercise training improves cardiorespiratory fitness and reduces cardiometabolic risk factors associated with the development of T2D. Accordingly, exercise training is consistently used as a model to examine putative mechanisms underlying insulin resistance and risk for T2D. To our knowledge, there are no studies that have examined the effect of exercise training on insulin resistance in obese black SA women who are at high risk for T2D. Therefore, the primary purpose of this research study is to gain a better understanding of the causal mechanisms underlying insulin resistance and risk for T2D in black SA women, using exercise as an intervention.

### Aims and Objectives

The aim of the study was to identify mechanisms underlying the changes in insulin sensitivity and secretion in response to a 12-week aerobic exercise intervention in obese black SA women.

The objectives of the study were as follows:

1. to measure changes in insulin sensitivity and secretion (primary outcomes) in response to the 12-week intervention compared with the nonexercise control group
2. to measure changes in potential primary and secondary determinants (secondary outcomes) of insulin sensitivity and secretion
3. to examine the associations between changes in insulin sensitivity and secretion and changes in the primary and secondary determinants

Primary determinants were as follows:

- cardiorespiratory fitness
- body composition and body fat distribution
- blood pressure
- lipid profile, including high-density lipoprotein (HDL)- and low-density lipoprotein (LDL)-cholesterol subclasses and HDL functionality
- ectopic fat deposition (skeletal muscle, liver, and pancreas)
- skeletal muscle expression of genes and proteins involved in insulin signaling, oxidative capacity, and mitochondrial biogenesis
- skeletal muscle and serum metabolomics and lipidomic profile
- gluteal and abdominal subcutaneous adipose tissue expression of genes and proteins involved in inflammation, insulin signaling, oxidative stress, vascularization, adipogenesis, and lipid metabolism
- circulating inflammatory cytokine, myokine, and adipokine concentrations
- skeletal muscle and adipose tissue mitochondrial respiration and hydrogen peroxide production;
- gastrointestinal microbiota

Secondary determinants were as follows:

- energy expenditure and substrate metabolism at rest and during exercise
- habitual physical activity and sedentary behavior patterns
- dietary intake, red blood cell, and adipose tissue fatty acid composition
- sleep quantity and quality
- psychological well-being
- perceptions of body image, healthy behaviors, and the exercise intervention

## Methods

### Study Design

**Figure 1:**
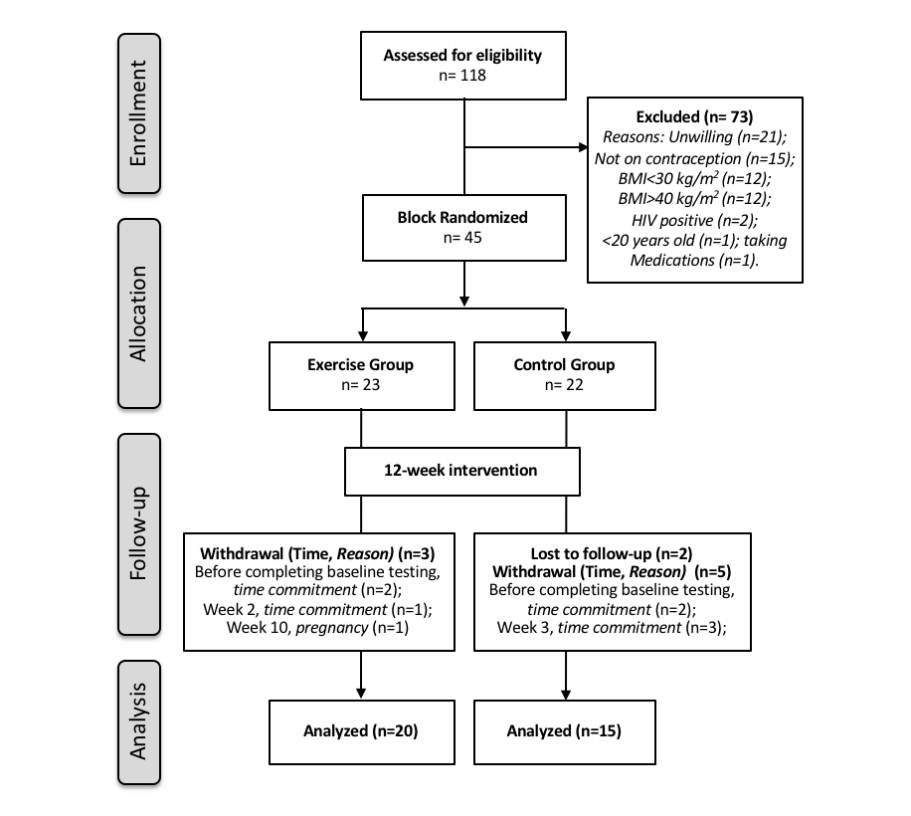
Consort diagram outlining participant enrollment, allocation, follow-up, and analysis. BMI: body mass index.

In this randomized controlled research study, 45 obese sedentary black SA women were block (2-4 participants) randomized (Microsoft Office, Excel, 2013) into control or experimental (exercise) groups (Figure 1). Block randomization was performed by the project manager after participants completed preintervention testing to ensure that investigators performing the testing were blinded to group allocation. The exercise group completed 12 weeks of supervised combined aerobic and resistance training (40-60 min, 4 days/week) but maintained their usual dietary behaviors. The control group were instructed to continue their habitual physical activity and dietary behaviors and to refrain from initiating any exercise programs. Before and following the 12-week intervention period (exercise or control), insulin sensitivity and secretion (primary outcome), as well as the proposed determinants of insulin sensitivity and secretion (secondary outcomes) were measured. In addition, dietary intake, sleep quality and quantity, physical activity, and sedentary behavior were monitored for a minimum of 4 days every 4 weeks.

This study was approved by the Human Research Ethics Committee at the University of Cape Town (HREC REF:054/2015). The study was performed in accordance with the principles of the Declaration of Helsinki (1964, amended last in Fortaleza Brazil, 2013), ICH Good Clinical Practice (GCP), and the laws of SA. Participants provided written informed consent before participation in the screening and the research study. Participant recruitment and testing procedures occurred over an 18-month period, between July 2015 and December 2016. Sample and data analysis began in January 2017 and are currently ongoing.

### Participants

Participants were recruited via advertisements in local papers and the distribution of flyers at local churches, universities and community groups in Cape Town, SA. Participants were included if they met the following inclusion criteria: (1) black SA women (based on the Xhosa ancestry of both parents) between the ages of 20 and 35 years; (2) obese (body mass index (BMI) 30-40 kg/m^2^); (3) weight stable (weight not changed more than 5 kg or no change in clothes size over the past 6 months); (4) sedentary (not participating in exercise training (>1 session of >20 min per week) within the last 12 months); (5) on injectable contraceptive (depot medroxyprogesterone acetate, 400 mg) for a minimum of 2 months; (6) no known metabolic or inflammatory diseases; (7) no hypertension (≥140/90 mmHg), diabetes (random plasma glucose concentration of >11.1 mmoL/L, and/or hemoglobin A_1c_ (HbA_1c_) result >6.5% A_1c_), HIV, or anemia (hemoglobin (Hb) <12 g/dL); (8) not taking any medications; (9) nonsmokers; (10) not currently pregnant or lactating; (11) no orthopedic or medical problems that may prevent exercise participation; and (12) no surgical procedures within the last 6 months.

### Screening

Before participation in the trial, volunteers completed screening procedures. Weight and height were measured to calculate BMI. Blood pressure was measured 3 times at 1-min intervals using an automated blood pressure monitor (Omron 711, Omron Health Care, Hamburg, Germany). A venous blood sample was drawn for the determination of glucose, Hb and HbA1_c_. HIV screening was performed and participants were excluded based on a confirmed positive test or if they refused to complete the test.

Participants received pre- and posttest counseling from a trained counselor, and a referral was made to appropriate HIV clinics for those participants who were found to be HIV-positive. Participants completed the physical activity readiness questionnaire (PARQ) [33] and were excluded if they answered “yes” to any of the questions. In addition, the participants completed a questionnaire about their exercise training, contraceptive use, ancestry, smoking status and history, medication use, and clinical conditions.

### Overview of Testing Procedures

**Figure 2:**
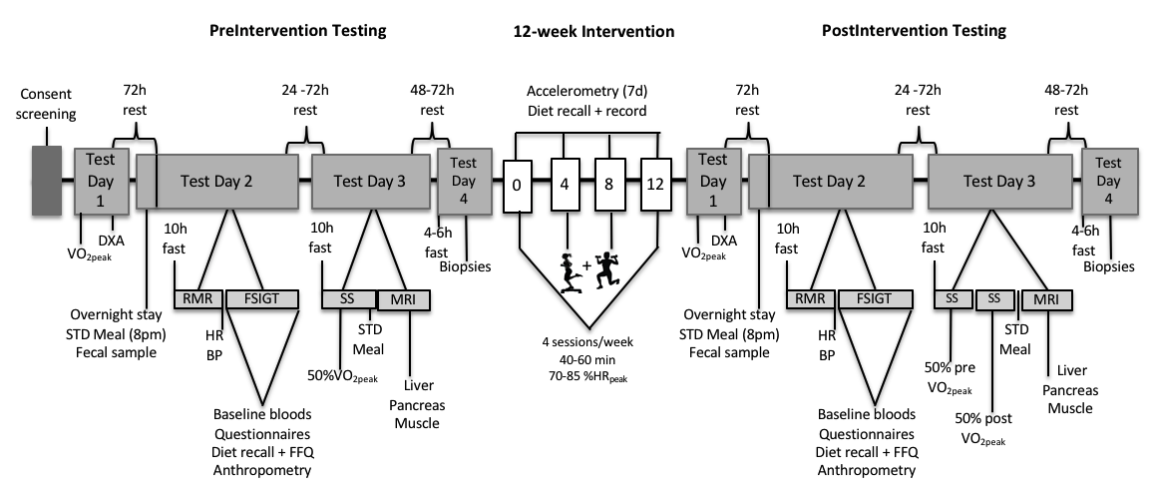
Schematic overview of testing timelines and procedures. VO2peak: peak oxygen consumption; STD: standard; DXA: dual-energy absorptiometry x-ray; RMR: resting metabolic rate, FSIGT: frequently sampled intravenous tolerance test; HR: heart rate; BP: blood pressure; FFQ: food frequency questionnaire; MRI: magnetic resonance imagery; SS: steady state treadmill test; %HRpeak: percent of peak HR.

The study design and stepwise stages of the protocol are described in Figure 2. Before and following the 12-week intervention, participants completed 4 data collection sessions. At the first session, participants completed a cardiorespiratory fitness test of peak oxygen consumption (VO_2peak_), and body composition was measured by dual energy x-ray absorptiometry (DXA). At least 72 hours later, the participants spent a night at the laboratory where they were given a standardized evening meal at 8 PM and then required to fast overnight (10 hours). During the evening or early morning, participants were requested to provide a fecal sample. At 6 AM, the participants completed measures of resting metabolic rate (RMR) and substrate metabolism, and resting heart rate and blood pressure were measured. At 7 AM, fasting blood samples were collected and participants underwent a frequently sampled intravenous glucose tolerance test (FSIGT). During the FSIGT, the field worker administered the questionnaires, and the participants completed a 24-hour dietary recall and food frequency questionnaire with a Health Professions Council of South Africa registered dietician. The participants were then requested to complete a 3-day food diary. On a separate day, after a 10- to 12-hour overnight fast, participants completed steady-state exercise testing at 50% VO_2peak_. Thereafter, participants were provided a standardized meal and underwent a magnetic resonance imaging (MRI) scan. On the fourth day of data collection, and after 4 to 6 hours of fasting and 48 hours of rest, participants underwent skeletal muscle and abdominal and gluteal SAT biopsies. Accelerometers (ActiGraph and ActivPAL) were attached to the participants and worn for 7 days.

Every 4 weeks following the start of the intervention, dietary intake (3-day dietary recall), physical activity and sleep quality and quantity (ActiGraph), and sedentary behavior (ActivPAL) were monitored. Following completion of the intervention, a subsample of women were invited to participate in focus group discussions and key informant interviews. Due to the large time commitments and travel requirements, participants were reimbursed at an hourly rate based on recommendations from the Health Sciences Human Research Ethics Committee of the University of Cape Town.

### 12-Week Exercise/Control Intervention

The exercise intervention consisted of 12-weeks of supervised aerobic and resistance training at a moderate-vigorous intensity for 40 to 60 min, 4 days per week by a trained facilitator. The exercise intervention was structured based on the results of a focus group study undertaken in the same community [34]. Exercises included cardiovascular exercises in the form of aerobic dance, running, skipping, and stepping that were performed at a moderate-vigorous intensity (75%-80% peak heart rate, HR_peak_). Resistance exercises included the participants using their own body weight and progressed to the use of equipment (eg bands and free weights). These exercises included squats, lunges, bicep curls, push-ups and shoulder press with a prescribed intensity of 60% to 70% HR_peak_. Attendance was recorded at each training session, and a heart rate monitor (Polar A300, Kempele, Finland) was worn by participants to ensure the prescribed exercise intensity was maintained throughout the 12-week period. Similarly, the respective resistance exercises were altered to ensure progression and to maintain the required intensity throughout the 12-week intervention. Training dose for the exercise group is calculated as the total number of sessions attended multiplied by the average percent of HR_peak_ attained over the 12-week period.

The control group was instructed to maintain their normal daily physical activity patterns, and not start any exercise training, which was verified through monthly monitoring using accelerometry. Following posttesting, the control participants were given the opportunity to participate in the 12-week exercise program, for which they were also reimbursed for their time and travel costs.

### Pre- and Postintervention Testing

#### Sociodemographic and Basic Health Information

The participants completed a demographic questionnaire that included measures of socioeconomic status (on the basis of factors such as asset index, education, housing and housing density, employment, and income) [35], family history of disease, personal health, reproductive history, supplement use, body image [36], alcohol use, and household food security [37]. In addition, measures of psychological well-being, including the Pittsburgh Sleep Quality Index [38], Beck Depression Inventory [39], the Kessler 10 [40], and the General Self-Efficacy [41] Questionnaires, were administered.

#### Body Composition Assessment

Basic anthropometry, including weight and height, in lightweight clothing without shoes, as well as waist circumference at the level of umbilicus, and hip circumference at the largest protrusion of the buttocks, were measured to the nearest 0.1 cm. Whole body composition, including fat mass and fat-free soft tissue mass (FFSTM), were measured by DXA (Discovery-W, software version 12.7.3.7; Hologic, Bedford, MA) according to standard procedures, with a coefficient of variation of 0.7% for FFSTM and 1.67% for fat mass. Subtotal (excluding the head) fat and FFSTM were used for all analyses. Regional body fat distribution, including arm, leg, trunk, gynoid, and android fat mass, was characterized as previously described [42] and abdominal VAT and SAT areas estimated [43].

#### Cardiorespiratory Fitness

To determine cardiorespiratory fitness, VO_2peak_ was measured using a treadmill-based (C, Quasar LE500CE, HP Cosmos, Nussdorf-Traunstein, Germany) graded exercise test. Participants were familiarized to the equipment before beginning the test, and heart rate was monitored throughout for the determination of HR_peak_ (Polar A300, Kempele, Finland). The initial 6 min of the test was designed based on a modified Bruce protocol to obtain three stages of steady state metabolism (see steady state protocol below) and the subsequent minutes were designed to obtain VO_2peak_ using a ramp protocol, adapted from Takagi et al [44]. Participants began at 3 km/hour at a 2% gradient for 2 min. The gradient increased by 2% for a further two 2-min segments. The following stages increased by 2% gradient every minute until 16%. Following this there was an alternate increase in speed (0.5 km/hour) and gradient (1%) until volitional exhaustion. This walking cardiorespiratory fitness test was designed for participants who were sedentary and are not familiar with gym-based equipment.

Pulmonary gas exchange was measured by determining O_2_ and CO_2_ concentrations and ventilation to calculate VO_2_ consumption using a metabolic gas analysis system (CPET, Cosmed, Rome Italy). Before each test, the gas meter was calibrated with a Hans Rudolph 3-liter syringe (Vacumed, Ventura, CA) and analyzers calibrated using standard gas mixtures of oxygen (26% O_2_ with the balance nitrogen) and carbon dioxide (4% CO_2_, 16% O_2_, and the balance nitrogen) (BOC Special Gas, Afrox Cape Town, South Africa). Ethanol burns for equipment calibration were conducted every 4 weeks (mean variance<2%).

#### Energy Expenditure and Substrate Metabolism During Submaximal Steady-State Exercise

Following an overnight fast (10-12 hours) respiratory exchange (Cosmed Quark CPET, Rome, Italy) was measured during 15 min of steady-state treadmill walking at 50% VO_2peak_, a level shown to be consistent with maximal fat oxidation [45,46]. Measures of energy expenditure and substrate metabolism were averaged over the last 10 min of the test. This test (50% pre-intervention VO_2peak_) was repeated at posttesting, which reflects the same absolute intensity. Participants then rested for 10 min and completed a second steady-state exercise test, at 50% of the postintervention VO_2peak_, which reflects the same relative exercise intensity.

#### Resting Metabolic Rate, Substrate Metabolism, and Blood Pressure

Participants slept overnight at the laboratory and were given a standardized evening meal at 8 PM (Energy: 2,456 kJ, 21 g protein (14% energy), 49 g carbohydrate (33% energy), and 32 g fat (48% energy). At 6 AM (following a 10-hour overnight fast), the participants rested in the supine position, in a quiet room (21°C -24°C), and were instructed to remain awake, still, and quiet. Basal respiratory exchange was measured for 40 min, using the ventilated hood technique (CPET, Cosmed, Rome Italy). The first 10 min were excluded to ensure measures of steady state respiratory gas exchange and the average of the last 30 min was used to determine resting measures. Weir [47] and Frayn [48] equations were used to calculate RMR and total rates of fat and carbohydrate oxidation, respectively. During the respective 40 min, the lowest recorded heart rate was recorded and reported as the resting heart rate.

#### Fasting Blood Samples and Frequently Sampled Intravenous Glucose Tolerance Test (FSIGT)

Following the RMR measures, fasting blood samples were drawn, and an FSIGT was performed. A cannula was inserted into a vein of each arm. One arm was used for intravenous glucose and insulin infusions, and the other arm was heated and used for blood sampling. Fasting blood samples were drawn for the determination of adipokines, myokines, inflammatory markers and cytokines, lipid profiles and HDL- and LDL-cholesterol subtypes and HDL functionality, and red blood cell fatty acid composition, metabolomic and lipidomic analysis.

For the FSIGT, further 2 baseline samples were collected at −5 and −1 min before a bolus of glucose (50% dextrose; 11.4 g/m^2^ body surface area) was infused intravenously over 60 seconds beginning at time 0. At 20 min, human insulin (0.02 U/kg; NovoRapid, Novo Nordisk) was infused over 5 min at a constant rate (HK400 Hawkmed Syringe Pump, Shenzhen Hawk Medical Instrument Co., Shenzhen, China). Samples for determination of plasma glucose and serum insulin and c-peptide concentrations were drawn at 2, 3, 4, 5, 6, 8, 10, 12, 14, 16, 19, 22, 23, 24, 25, 27, 30, 35, 40, 50, 60, 70, 80, 90, 100, 120, 140, 160, 180, 200, 220, and 240 min.

Fasting blood samples were collected into EDTA, lithium heparin, fluoride oxalate, and SST tubes. Samples in SST tubes clotted for 30 min at room temperature, while the remaining samples were placed on ice before centrifugation. Samples were centrifuged at 3000 rpm for 10 min at 4°C. Plasma for glucose analysis was stored at −20°C, while the remaining serum and plasma was stored at −80°C. Red blood cells collected from EDTA tubes were washed by 2 cycles of sequential centrifugation at 1000 rpm and a final cycle of centrifugation at 3000 rpm for 10 min. Between cycles, saline (0.9% NaCl in distilled water) was used for resuspension and washing. Red blood cells were then stored at −80°C until the analysis.

Glucose and insulin concentrations from the FSIGT will be used to calculate the insulin sensitivity index (S_I_) using Bergman’s minimal model of glucose kinetics [49]. Glucose and c-peptide data will also be used in a two-compartment minimal model of C-peptide secretion and kinetics to calculate insulin secretion rate (ISR) using WinSAAM (version 3.3.0). ISR will then be used in a one-compartment insulin minimal model to determine insulin hepatic extraction index [50].

#### Fecal Sample Collection

Participants provided fecal samples for the analysis of gastrointestinal microbial composition using the Easy Sampler stool collection kit (EasySampler, GP medical devices, Denmark), as per the manufacturer’s instructions. The samples were immediately stored at −80°C, until subsequent analysis.

#### Ectopic Lipid Content

After the steady-state exercise test, the participants consumed a standardized meal (Energy: 2553 kJ; protein: 20.9 g; carbohydrates: 83.0 g; fat: 22.2 g). Hepatic, pancreatic, and skeletal muscle (tibialis anterior and soleus) lipid content were then measured on a 3-Tesla Skyra wholebody human MRI scanner (Siemens, Erlangen). Sequence protocols for fat assessments included MRI using two-point Dixon fat-water separation (Dixon-VIBE) and T1-VIBE with and without fat saturation, and finally, MRS with PRESS technique.

Postprocessing of MRI data for Dixon and T1-VIBE was performed in MATLAB R2009a (MathWorks Inc, Natick, MA, USA). The MRS voxel locations were coregistered to T1-VIBE images (with and without fat suppression) and Dixon images (fat and water images) to compute the fat fraction. The signal fat-fraction was calculated as the signal without fat suppression minus signal with fat suppression divided by signal without fat suppression for the T1-VIBE method [51]. The signal fat fraction obtained by the Dixon method was calculated by combining images obtained from the water and fat phases, as the fat fraction divided by the fat plus water fractions [52]. MRS data were quantified using LCModel (version 6.3-1J) **.** The MRS method was used to decompose the lipid signals into several components, each one representing different parts of the lipid metabolite molecules. The lipid signals were reported relative to the water signal.

#### Skeletal Muscle and Adipose Tissue Biopsies

After a 4- to 6- hour fast and at least 48 hours after exercise, fat and muscle samples were collected. Fat samples were obtained from the gluteal and abdominal SAT depots using a mini-liposuction technique [16]. After local anesthesia with Lignocaine hydrochloride (2%, Intramed, Port Elizabeth, South Africa), a small incision was made into the region of interest and 200 mL of normal saline with 20 mL 2% Lignocaine (Intramed) was infused using an infiltration cannula (Lamis 14 ga x 15 cm, Byron Medical Inc., Tucson, AZ, USA). An aspiration cannula (Coleman, 12 ga x 15 cm, Byron Medical Inc.) attached to a 10-mL syringe was used to aspirate fat. Using this procedure, abdominal samples were obtained from directly above the umbilicus, and gluteal samples were obtained from the right upper outer quadrant. Approximately 2 cm^3^ to 3 cm^3^ of fat was extracted from each site and washed 3 times with normal saline or until no blood was visible. A subsample of the adipose tissue was placed in ice-cold BIOPS for immediate analysis of mitochondrial respiration. The remaining adipose samples were placed into vials and frozen immediately in liquid nitrogen (N_2_) and stored at −80°C for the analysis of gene and protein expression, and fatty acid composition. After local anesthesia (2%, Intramed), a skeletal muscle biopsy was taken from the *M vastus lateralis* muscle using a 5-mm Bergstrom needle according to the needle biopsy technique of Bergstrom [53]. A subsample was placed in ice-cold BIOPS, for immediate analysis of mitochondrial respiration. The remaining samples were immediately frozen in liquid N_2_ and stored at −80°C for subsequent analysis of gene and protein expression, as well as metabolomics and lipidomics.

### Monthly Monitoring

#### Physical Activity, Sedentary Behavior, and Sleep

Physical activity and sleep quality and quantity were measured using accelerometry (ActiGraph GTX3+, ActiGraph LLC, Pensacola, Florida), and sedentary behavior was measured using activPAL (activPAL3c, PAL Technologies Ltd, Glasgow, UK) at preintervention, week 4, week 8, and postintervention. The ActiGraph was initialized to record data in 60-second epochs and was set to measure motion in all 3 axes, with the inclinometer function activated. The ActiGraph was worn on the right hip with a lightweight belt, and participants were instructed to wear it for 24 hours a day over a 7-day period, except when swimming, bathing, and showering. Participants were instructed to complete a sleep diary to capture awake and sleep times. Physical activity and sleep data were captured and analyzed using the ActiLife Software Version 6 (ActiLife software; Pensacola, FL, USA). A minimum of 4 days of wear time, with 600 min per day of wake time was required for data analysis. The 4 days of wear needed to be inclusive of 3 weekdays and 1 weekend day. For the exercise group, at least one of the weekdays needed to be an “exercise day.” Nonwear time was defined as 60 continuous minutes of no counts (zeros) [54]. Vector magnitude cut-points were used for analysis [55,56]. The vector magnitude represents the summed value of all 3 axes measured from the ActiGraph, calculated as the square root of the total sum of each axis, squared (X^2^+Y^2^+Z^2^), then square rooted [57]. Counts/minute between 200 and 2689 represents light intensity physical activity, 2690 to 6166 represents moderate intensity physical activity, 6167 to 9642 represents hard intensity physical activity, and >9643 counts per minute represents very hard intensity physical activity. Data were analyzed for any physical activity occurring in 1-min and 10-min bouts/intervals. Within each 10-min bout, 1- or 2-min of “dropped” counts were allowed, thereby excluding bouts of activity where a drop-in count is greater than 2 min (within the 10-min period) occurred. Sleep data were analyzed for sleep latency, total sleep time, wake after sleep onset, and sleep efficiency. Participants completed a sleep diary that was used to mark wake and sleep hours, which was further verified based on movement measured by the activPAL.

The activPAL was attached to the midanterior right thigh using a waterproof sleeve and dressing and worn concurrently with the ActiGraph, without removal of the device, even during bathing or swimming. All data were downloaded using the activPAL software (PAL Technologies, version 7.2.32, Glasgow, UK), and event files were used to create second-level files to show time spent in sitting (or lying), standing, stepping, sit-to-stand transition, and stand-to-sit transition.

#### Dietary Intake

At the same time points as the physical activity data collection, dietary intake was estimated using a 24-hour recall and a 3-day dietary record, including 2 weekdays and 1 weekend day. In addition, a food frequency questionnaire was administered before and following the intervention. Nutrient intake and food group analysis were calculated using the South African Food Composition Database System (SAFOOD, the South African Food Composition Database, South African Medical Research Council, Cape Town, South Africa).

#### Perceptions of the Exercise Intervention

Focus group discussions (FGDs) and in-depth interviews were used to explore the perceptions participants had of the exercise intervention. A multiple-category qualitative research design was applied in this study [58]. This type of design includes conducting focus groups with different types of participants either sequentially or simultaneously [58]. This approach ensures a comparison from one group to another within a category and/or from one category to another category [58].

The focus group interview schedule included questions such as “What are some of the things that influenced your attendance to the exercise sessions?”, whereas the in-depth interviews included 1 main open ended question aimed at obtaining the participants’ experience of the exercise sessions (Multimedia Appendix 1). The latter were conducted after the FGD and participants who were the most vocal during the group discussions were purposively selected for the in-depth interviews.

Four FGDs were conducted (3-5 participants per group), including exercise participants, and control participants who had chosen to participate in the exercise sessions upon completion of the 12-week intervention. The group discussions were moderated by a trained facilitator, fluent in isiXhosa, which is the language predominantly spoken by the participants. The audio recording of the FGDs was translated and transcribed by a trained professional. Immediately after completion of the FGDs, the researcher and moderator identified participants to be invited to participate in the in-depth interviews. A total of 5 in-depth interviews were conducted **.** Thematic analysis was used to determine the salient themes that emerged during the FGDs using Atlas.ti Qualitative Data Analysis Software (Scientific Software Development GmbH, Berlin, Germany) [59].

### Biochemical Analysis

#### Glucose, Insulin, C-Peptide, and Lipid Profile

Plasma glucose and serum lipids concentrations were determined using a colorimetric assay (Randox, Gauteng, South Africa) and serum insulin, C-peptide were measured using immunochemiluminometric assays (IMMULITE 1000 immunoassay system, Siemens Healthcare, Midrand, South Africa).

#### Serum Inflammatory and Oxidative Stress Markers

Inflammatory cytokines, including interleukin (IL)6, IL1R, IL8, IL10, monocyte chemotactic protein (MCP)1, IL15, interferon (IFN) gamma, and tumor necrosis factor (TNF) alpha, were measured using Milliplex MAP MAG Human Cytokine kit (Merck, Johannesburg, South Africa) and xMAP technology (Luminex, Austin, Texas) according to the manufacturer’s instruction. Serum concentrations of leptin and high molecular weight (HMW) adiponectin (EMD Millipore Corporation, St Charles, Missouri, USA) were analyzed using commercially available ELISA kits according to the manufacturer’s protocols. High-sensitive C-reactive protein (CRP) was measured by an immunochemiluminometric assay (IMMULITE 1000 immunoassay system, Siemens Healthcare, Midrand, South Africa). Lipid peroxidation was assessed by measuring the concentration of thiobarbituric acid reactive substance (TBARS); antioxidant capacity was assessed by measuring oxygen radical capacity absorbance (ORAC), as well as catalase and superoxide dismutase (SOD) activities as described previously [60].

#### Red Blood Cell and Adipose Tissue Fatty Acid Composition

Total lipids of red blood cell aliquots (RBC; 300 µL) and adipose tissue portions (100 mg) were extracted (2:1; v:v; chloroform:methanol containing 0.01% butylated hydroxytoluene) by using a modification of the Folch et al method [61,62]. Red blood cell total phospholipid fatty-acid (FA) and adipose tissue total FA percentage composition were determined by gas-liquid chromatography as previously described [62]. Pairwise analysis of gluteal and abdominal samples was performed including the pre- and postsamples of a participant in the same batch on the same day. Product to precursor FA ratios were used as a proxy to reflect delta-6- and delta-5-desturase enzyme activity [63].

#### Comprehensive Metabolite Profiling of the Serum and Muscle Metabolome

For the metabolite analyses, a combined platform of liquid (LC-QTOF-MS) and gas (GCTOF-MS lipids) chromatography coupled to mass spectrometry, in both positive and negative ionization modes will be used. This approach will enable a comprehensive coverage of serum and muscle metabolites with different chemical properties. All sample preparation and analyses will be performed according to a run order design to circumvent methodological biases that may interfere with results interpretation [64]. For example, samples from the same individual are prepared and analyzed in close connection while keeping the internal sample order randomized. Analytical batches will be balanced in terms of treatment group and quality control (QC) samples (ie, pooled from all samples) will be analyzed continuously.

Serum samples will be prepared according to A et al [65], using a 90/10, v/v methanol:water extraction including internal standards for metabolomics; and a 70/30, v/v chloroform:methanol extraction for lipidomics [66].

On an average, we will detect 2000 to 3000 peaks or more, and annotation/identifications will be done via the use of publically available library, in combination with *in house* library at the Swedish Metabolomics Centre (SMC). For targeted analyses and validation of findings, we will use triple quadrupole mass spectrometry techniques, such as LC-QqQ-MSMS or GC-QqQ-MSMS together with LC-TQMSMS and GC-TQMSMS in MRM-mode. Absolute quantification of specific compounds will be achieved by using calibration curves calculated from stable isotope labeled internal standards.

### Serum HDL- and LDL-Cholesterol Subclasses and HDL Functionality

HDL was isolated from aliquots of serum using density shift ultracentrifugation as described previously [67,68]. HDL anti-inflammatory function was measured by assessing expression levels of vascular cell adhesion molecule (VCAM) human umbilical vein endothelial cells (HUVEC) treated with participant HDL and stimulated with murine TNF-α, as described previously [67]. HDL antioxidant function was quantified by measuring serum paraoxonase-1 (PON1) activity as described previously [67]. HDL-induced reverse cholesterol efflux was quantified using a modified method [69]. Briefly, RAW264.7 cells (Gill Dealtry, Nelson Mandela Metropolitan University), were labeled with [^3^H] cholesterol in a medium containing acyl-CoA cholesterol acyltransferase (ACAT) inhibitor. Isolated participant HDL was then added and cholesterol efflux was carried out for 4 hours. Reverse cholesterol efflux capacity was calculated as label present in the cell media relative to the untreated control. HDL anti-thrombotic function was quantified by measuring serum platelet activating factor acetylhydrolase (PAF-AH) activity using the PAF Acetylhydrolase Assay Kit (Cayman Chemical, 760901). Serum HDL and LDL subclass were determined using the Lipoprint HDL and LDL systems (Quantimetrix, Redondo Beach, CA) as described previously [67,70].

### Skeletal Muscle and Adipose Tissue Gene and Protein Expression

RNA was extracted from adipose tissue and skeletal muscle samples using RNeasy Mini lipid kit (Qiagen Ltd, Germantown, MD, USA) and mirVana miRNA Isolation kit (Invitrogen, Life technologies, Carlsbad, CA, USA), respectively. Skeletal muscle RNA was DNAse treated using DNA-free Kit (Invitrogen, Life technologies, Carlsbad, CA, USA). RNA was reverse transcribed to cDNA using the High-Capacity cDNA Reverse Transcription Kit with RNase inhibitors (Applied Biosystems Foster City, CA, USA).

For the adipose tissue, RT-PCR will be performed in triplicate using Applied Biosystems QuantStudioTM 3 Real-Time PCR system with predesigned Taqman assays from Applied Biosystems (Warrington, UK) (see Multimedia Appendix 2). The genes of interest will be measured and presented as the ratio of abundance of the gene of interest: mean of abundance of the relevant housekeeping genes (LRP10 and RPLPO). Protein expression, and phosphorylation status, for genes of interest will be analyzed using ELISA and/or Western Blot analyses.

For skeletal muscle, a gene array was conducted using Human Affymetrix Cartridge Clariom S Platform (Affymetrix, Santa Clara, CA, USA) and analyzed with Affymetrix Expression Console using the SST-RMA method. Unlogged signals were compared using a 2-tailed paired Student *t*-test. The q-values (false discovery rates) were calculated by the R/Bio-conductor function and set at q<.05. Target and novel pathways will be investigated using the gene array data, and genes of interest and associated proteins will be further validated using RT-PCR and western blots analyses, respectively.

### Skeletal Muscle and Adipose Tissue Mitochondrial Respiratory Function

Measures of mitochondrial respiration were performed in respiration medium (MiR05) at 37°C using high-resolution Oxygraph-2k (Oroboros, Innsbruck, Austria) [71]. All measures were completed in duplicate and carried out in a hyperoxygenated (250-450 nmoL/mL) environment. Skeletal muscle and adipose tissue (abdominal and gluteal subcutaneous adipose tissue) samples were prepared and analyzed according to the methods described [71,72]. Briefly, immediately after tissue collection, samples were stored in ice-cold BIOPS [71] for a maximum of 4 hours before analyses. Skeletal muscle fibers (1-3 mg w/w) and adipose tissue (50-60 mg w/w) were permeabilized in saponin (5 mg/mL BIOPS) for 30 min and 20 min, respectively. Tissue was immediately washed in MiR05 for 2 x 10 min. The multiple substrate-uncoupler-inhibitor titration protocol applied to all tissue is as follows [73,74]:

1. Medium chain fatty acid oxidation through leak respiration in the absence of adenylates with the addition of malate (2 mM) and octanoly-carnitine (0.2 mM)
2. Maximal flow of electrons through electron transferring flavoprotein and fatty-acid oxidation (ADP 5 mM)
3. Submaximal state 3 respiration capacity specific to complex I (pyruvate 5 mM; glutamate 10 mM)
4. Maximal state 3 respiration, oxidative phosphorylation capacity (Succinate, 10 mM)
5. State 4o respiration, oligomycin-induced leak respiration through inhibition of ATP synthase (Oligomycin 2.5 μM)
6. Electron transports system capacity with the titration of CCCP (0.5 μM titration steps)
7. Inhibition of complex I with the addition of rotenone (0.5 μM)
8. The inhibition of complex III with the addition of antimycin A (2.5 μM)

Complex III inhibition was used for the determination and correction of residual oxygen consumption (nonmitochondrial oxygen consumption in the chamber). Ascorbate (2 mM) and TMPD (0.5 mM) were added to assess cytochrome c oxidase (COX), complex IV activity. TMPD and ascorbate are redox substrates that donate electrons directly to COX, and activity was measured by pmol of O_2_ a minute per mg of wet weight.

Hydrogen peroxide (H_2_ O_2_) flux was measured simultaneously with respirometry in the O2k-Fluorometer (O2k-Fluo LED2-Module Fluorescence-Sensor Green) using the H_2_ O_2_ sensitive probe Amplex UltraRed. Then 10 μM Amplex UltraRed and 1 U/mL horseradish peroxidase (HRP) was added to the chamber. The reaction between Amplex UltraRed and H_2_ O_2_ catalyzed by HRP is fluorescent, similar to resorufin. Calibrations were performed throughout the respirometry experiment to account for degradation of fluorescent over time, with 2 steps of H_2_ O_2_ added at 0.1 μM per step. Mass-specific H_2_ O_2_ were calculated relative to oxygen flux (H_2_ O_2_/O_2_ flux). The H_2_ O_2_/O_2_ flux ratio is frequently applied to evaluate the relative importance of H_2_ O_2_ production at different respiratory states [75].

### Fecal Bacterial Community Analysis

Bacterial DNA will be extracted using the ZymoBIOMICS DNA Miniprep kit (Zymo Research Corp., Irvine, USA) according to the manufacturer’s instructions. Bacterial composition will be described by sequencing the V4 hypervariable region of the 16S ribosomal RNA gene. Sequencing libraries will be prepared as per the Illumina MiSeq system instructions (Illumina, San Diego, CA). The pooled library will then be sequenced on the Illumina Miseq sequencing platform (Illumina, San Diego, CA). Raw sequences obtained from the Illumina Miseq will be subjected to a quality check using the FastQC software [76]. Preliminary analysis of the raw data will involve removing primers, barcodes, contaminants, and low-quality bacterial sequences. The 16S pair-end reads will be assembled by joining forward and reverse of demultiplexed sequence reads. The output file will then be processed for quality filtering. Chimeric sequences will be filtered by UCHIME algorithm in USEARCH platform. We will use QIIME [77] to cluster sequences into operational taxonomic units (OTUs) based on a sequence similarity threshold of 97%. The SILVA database will be used to assign taxonomic identities to the OTUs. Moreover, we will use the PICRUSt (Phylogenetic Investigation of Communities by Reconstruction of Unobserved States) and BugBase softwares to predict the metabolic function [78,79]. Raw data in fastq format will be made available in a public sequence database.

### Statistics

#### Sample Size Determination

Sample size determination was based on our primary outcome using the study of Nordby et al [80], with a significance level of *P*<.05 and power of 80%. On the basis of the change in normalized glucose clearance (measured using a euglycemic hyperinsulinemic clamp) from pre- to postintervention (12-week aerobic training) compared with the nontrained control group (8.2 [SD 5.9] vs −1.8 [SD 6.2] mL/kg/min FFM/nmoL/L insulin, respectively), 6 participants per group would be required to detect a significant difference between groups. These numbers correspond to those of Ortega et al [81] who compared glucose tolerance tests to detect the insulin sensitizing effects of a bout of continuous exercise and reported that 6 participants would be required to detect a change in insulin sensitivity when using a FSIGT. In order to account for the secondary outcomes, using changes in skeletal muscle glucose transporter (GLUT)4 in response to a 12-week training program as the proxy (0.65 [SD 0.69] vs 0.01 [SD 0.69] AU for training vs control group), 18 women would be required to detect a difference between groups [81]. On the basis of these calculations, and a dropout rate of 10% (2/20), 20 participants per group were selected.

#### Proposed Statistics

Results will be presented as means (SD), or medians and interquartile ranges for non-normally distributed data. Data will be normalized by log transformation if required. Repeated measures ANOVA will be used to compare differences in the change of outcome variables between the control and exercise groups in response to the exercise/control intervention. Pearson and/or Spearman correlations will be used to examine associations between changes in primary and secondary outcome variables. Data will be analyzed using StataSE (version 14, StatCorp, Texas, USA) and IBM SPSS Statistics 24 (Version 24.0, Armonk, NY, USA).

Comprehensive metabolite profiling data will be evaluated via a combination of multivariate analysis methods and univariate statistics, that is, Principal Component Analysis (PCA), Orthogonal Partial Least Squares (OPLS), and its extensions [64]. In addition, we will perform an extensive validation to determine the model significances, using both internal, ANOVA on the cross-validated model patterns, and external validation, independent sample prediction. Model scores (subject level) will be used to visualize and interpret the differences in metabolic response between predefined patient groups or subgroups, or of individual patients responding differently to the interventions. Model loadings (variable level) combined with univariate *P* values will be used as the base for mechanistic interpretation and to highlight significant metabolites or metabolite patterns.

For bacterial community analysis, basic statistical tests will be performed using QIIME [77] and R software will be used for advanced statistical analysis. Beta-diversity will be evaluated by calculation of weighted and unweighted Unifrac distances. The Shannon and Simpson diversity indices will be employed to study alpha-diversity. The relationship between the composition of the fecal bacteria and exposure variables will be determined using weighted generalized ridge regression methods [82] and the lasso [83]. We will use Dirichlet multinomial models or ecological approaches such as multi-species occupancy models to evaluate interactions and shift in fecal bacterial communities over time [84,85].

## Results

### Participant Enrollment, Allocation, Follow-Up, and Analyses

Information on participant enrollment, allocation, follow-up, and analysis is shown in Figure 2. Standards of reporting were based on the CONSORT 2010 checklist for randomized control trials. A total of 45 participants were enrolled in the study and randomized into exercise (n=23) and control (n=22) groups. Of these, 10 participants did not complete the intervention (dropout; n=3 exercise, n=7 control) resulting in a final sample of 20 exercise and 15 control participants.

### Participant Baseline Characteristics

The sociodemographic characteristics of the participants at baseline are presented in Table 1. The average age of the whole group (n=45) was 24 (SD 4) years. Sociodemographic characteristics were not different between participants of the exercise, control, and dropout groups. The majority (67%, 30/45) of participants had completed at least grade 12 education, and 27% (12/45) of participants were currently enrolled students, while 51% (23/45) were employed. A quarter of the participants earned less than R2500/month (US $210/month at exchange rate of R11.9/US $, 13 February 2018), whereas 42% (19/45) earned between R2500-R5000/month (US $210-420/month), and the remaining participants (31%, 14/45) earned greater than R5000/month (US $420/month). Most of the participants were not married (89%, 40/45) and 42% (23/45) had at least 1 child. Apart from hypertension (42%, 19/45) and diabetes (13.6%, 6/45), the known family history of disease was relatively low.

**Table 1:** Baseline sociodemographic characteristics.

Variables		Control (n=15)	Exercise (n=20)	Dropout (n=10)	*P* value
Age in years, mean (SD)		24 (4)	23 (3)	26 (4)	.24
Informal housing, n (%)		6 (40)	4 (20)	6 (60)	.09
Housing density (persons/room), median (interquartile range)		1.3 (1.0-1.6)	1.0 (0.8-1.5)	1.0 (1.0-2.7)	.38
Asset index (% of 14 commodities), n (%)		53 (15)	54 (20)	47 (23)	.62
**Education, n (%)**					.87
	< Grade 12	6 (43)	5 (25)	3 (30)	
	Grade 12	5 (36)	9 (45)	4 (40)	
	Tertiary	3 (21)	6 (30)	3 (30)	
**Employment, n (%)**					.63
	Employed	9 (60)	10 (50)	4 (40)	
	Student	5 (33)	9 (45)	4 (40)	
	Unemployed	1 (7)	1 (5)	2 (20)	
**Income, n (%)**					.09
	R0-2499/month	6 (40)	2 (10)	4 (40)	
	R2500-R4999/month	7 (47)	8 (40)	4 (40)	
	>R5000/month	2 (13)	10 (50)	2 (20)	
Marital status, married, n (%)		1 (7)	3 (15)	1 (10)	.73
**Parity, n (%)**					.33
	None	7 (48)	15 (75)	4 (40)	
	1 child	4 (27)	2 (10)	3 (30)	
	2-3 children	4 (27)	3 (15)	3 (30)	
**Known family history of disease, n (%)**					
	Hypertension	7 (47)	9 (45)	3 (30)	.38
	Heart disease	0 (0)	3 (16)	1 (10)	.63
	Stroke	1 (7)	1 (5)	1 (10)	.67
	Diabetes	2 (13)	2 (11)	2 (20)	.74
	Obesity	0 (0)	1 (5)	0 (0)	.66

The baseline cardiorespiratory fitness, physical activity, and dietary intake did not differ between groups (Table 2). For the whole group, despite cardiorespiratory fitness being low (<25 mL/kg/min), the participants accumulated an average of 9338 steps per day. However, most of the day was spent in sedentary behavior (54%). The majority of dietary energy intake (8390 [6577.0-9540.0] kJ/day) was derived from carbohydrate (56.3%, 7.0%), followed by fat (29.4%, 7.0%) and then protein (14.0%, 2.6%). Dietary sugar intake was high (64.7 [51.5-108.6] g/day), and fiber intake below recommendations for adequate intake (17.0 [14.3-23.2] g/day vs recommendations of 25 g/day). 

The baseline anthropometry and cardiometabolic risk factors of the participants are presented in Table 3. Body composition did not differ between groups. For the whole group, the mean BMI, waist and waist to hip ratio were 33.9 (SD 2.8) kg/m^2^, 103.8 (SD 8.0) cm and 0.90 (SD 0.07), respectively. Cardiometabolic risk factors did not differ by group. The participants were all normotensive (systolic: 110.1 [SD 10.7] mm Hg; diastolic: 73.1 [SD 9.0] mmHg) and had normal glucose tolerance based on HbA_1c_ (5.2 [SD 0.3] A_1c_%).

**Table 2:** Baseline cardiorespiratory fitness, physical activity and dietary intake.

Variable		Control	Exercise	Dropout	*P* value
**Cardiorespiratory fitness, mean (SD)**					
	n	15	20	9	
	VO_2peak_^a^ (mL/min)	2099 (281)	2077 (211)	1989 (296)	.55
	VO_2peak_ (mL/kg/min)	23.9 (3.0)	24.9 (2.4)	23.4 (4.5)	.43
**Physical activity (ActivPAL), mean (SD)**					
	n	15	19	7	
	Steps (No. Day)	10013 (2650)	9349 (2334)	7858 (2756)	.19
	Stepping (% day)	14 (4)	12 (3)	11 (3)	.19
	Standing (% day)	34 (9)	34 (8)	32 (8)	.83
	Sedentary (% day)	52 (10)	54 (9)	58 (9)	.48
**Dietary intake, median (interquartile range) or mean (SD)**					
	n	15	20	10	
	Energy intake (kJ/day)	8138 (6493-9434)	8966 (7119-11,775)	8921 (7875-9396)	.45
	Fat (%EI^b^)	30.9 (5.6)	30.3 (6.2)	27.1 (7.1)	.32
	Protein (%EI)	14.3 (1.9)	13.1 (5)	14.2 (3.6)	.37
	Carbohydrate (%EI)	54.1 (.7)	55.3 (5.8)	57.9 (8.4)	.38
	Sugar (g/day)	64.7 (35.3-108.0)	62.5 (54.3-92.1)	75.8 (53.9-130.8)	.57
	Fiber (g/day)	16.2 (11.9-23.2)	18.9 (15.1-24.4)	17.0 (10.4-23.7)	.51

^a^VO_2peak_: peak oxygen consumption.

^b^%EI: percentage of total energy intake.

**Table 3:** Baseline anthropometry and cardio-metabolic risk factors.

Variable		Control	Exercise	Dropout	*P* value
**Anthropometry, mean (SD)**					
	n	15	20	10	
	Height (m)	1.62 (.06)	1.57 (.06)	1.60 (.06)	.05
	Weight (kg)	87.8 (10.9)	84.1 (8.7)	87.5 (12.0)	.52
	BMI^a^ (kg/m^2^)	33.4 (2.7)	34.1 (2.8)	34.1 (3.3)	.72
	Waist (cm)	103.4 (8.1)	103.6 (7.4)	106.8 (11.9)	.75
	WHR^b^	0.88 (0.05)	0.91 (0.07)	0.90 (0.08)	.45
**Cardio-metabolic risk factors, mean (SD)**					
	n	15	20	4	
	Systolic BP^c^ (mmHg)	111.7 (11.3)	109.0 (11.1)	109.5 (8.0)	.76
	Diastolic BP (mmHg)	75.0 (9.7)	72.2 (9.4)	70.8 (1.5)	.57
	HbA_1c_^d^	5.2 (0.4)	5.2 (0.3)	5.2 (0.1)	.90

^a^BMI: body mass index.

^b^WHR: waist to hip ratio.

^c^BP: blood pressure.

^d^HbA_1c_: hemoglobin A_1c_.

## Discussion

This is the first study, to our knowledge, that has used an exercise intervention to understand the mechanisms underlying the high risk for T2D in a black African population. The study uses state-of-the-art technology to characterize the determinants of insulin sensitivity and secretion. Using exercise as a model ensures a holistic approach that focuses on the complex interaction of biological systems, within the context of associated environmental and lifestyle factors. Novel biological aspects of the protocol in this cohort include (1) measurement of pancreatic fat content by MRS; (2) an array approach in the skeletal muscle to identify novel pathways and genes involved in the regulation of insulin sensitivity, in combination with metabolomic and lipidomic analyses; and (3) characterization of the gastrointestinal microbiome. Although the aim of the study was to understand the primary and secondary determinants of insulin sensitivity, the study results, as well as the findings of the focus group discussions and key informant interviews can be used to inform the suitability of this intervention for large-scale roll out in similar communities.

The participants were a homogenous cohort of young women of Xhosa ancestry who mostly resided in an informal urban settlement and were of a low socioeconomic status. Notably, 38% (17/45) of the participants were meeting physical activity guidelines by accumulating more than 10,000 steps/day [86]. As shown in our previous research [31], these steps are usually accumulated through walking for transport that is typically conducted at a low intensity [32], which is subsequently reflected in the participants low cardiorespiratory fitness levels (VO_2peak_) and a rating of “poor,” according to the American College of Sports Medicine [33]. Accordingly, exercise training at a moderate to high intensity offers an ideal intervention within this population to ensure improvements in cardiorespiratory fitness and associated cardiometabolic outcomes, specifically insulin sensitivity. It is anticipated that the exercise training will improve insulin sensitivity and reduce the risk for T2D within this high-risk population.

Although 118 women were screened, only 45 women were willing and eligible to be enrolled in the study. A requirement for screening was to consent for HIV testing, which deterred many potential participants from screening and is likely related to the high rates (20%) of HIV in women in the City of Cape Town [87]. The main reasons for ineligibility after screening included (1) not meeting the BMI criteria, (2) not using injectable contraception, and (3) time limitations and/or the invasiveness of procedures involved in the study. The injectable contraception was chosen as part of the selection criteria to ensure a more homogenous participant profile. The injectable contraception is freely available to all women in the community clinics and is thus the contraception of choice for the majority of women. Of the 45 women recruited, 35 completed the 12-week intervention, with the greatest dropout being in the control group (7 out of the 10 participants; Figure 1). This may be explained by the control groups’ disappointment on not being assigned to the exercise group, resulting in a lack of commitment and loss of interest in the study. This occurred despite the assurance that the control group could participate in the 12-week exercise intervention following the 12-week control period, for which they would be reimbursed for their time, inconvenience, and travel. Interestingly, only 9 of the 15 control participants initiated the exercise training, of which 6 attended more than 30 of the 48 training sessions, and 3 participants attended less than 3 sessions. In contrast, there was a low dropout rate in the exercise group (3 out of the 10 participants). It is anticipated that the outcomes from the focus group and informant interviews will provide insight regarding the reasons for the discrepancies in the dropout rates between the groups.

There are several strengths to this exercise intervention that involve, among others, monitoring and collaboration. First, every exercise session was facilitated by a trained exercise physiologist, who ensured that the prescribed exercise intensity was attained by using heart rate monitoring and ratings of perceived exertion. Moreover, changes in lifestyle factors, including dietary intake (red blood cell and adipose tissue fatty acid composition), sleep, physical activity, and sedentary behavior (accelerometry) were objectively monitored every 4 weeks over the 12-week intervention. Finally, the collaborative nature of the study ensures the incorporation of diverse skills and expertise from both local (South African) and international (Sweden and USA) collaborators. This allows for a systems biology approach to understand the mechanisms underlying the high risk for T2D in an African population. However, the sample size is limited due to the costs associated with the extensive testing and time and commitment requirements from the participants. Nevertheless, the study is powered for the main outcome measures, and partial least squares regression will be used due to its capacity to deal with very small sample sizes and many parameters [64]. Furthermore, the data from the secondary outcomes may also be used as pilot data to inform future studies.

In conclusion, we have described a research protocol for an exercise intervention to understand the mechanisms underlying insulin sensitivity and secretion in obese black SA women. The knowledge gained from this study will be used to inform future interventions and treatments to combat insulin resistance and T2D in this high-risk population.

## Appendix

Multimedia Appendix 1 Focus group discussion open-ended guide questions.

Multimedia Appendix 2 Adipose tissue genes of interest.

## Abbreviations

DXA: dual energy X-ray absorptiometry
FA: fatty-acid
FFSTM: fat-free soft tissue mass
FGDs: focus group discussions
FSIGT: frequently sampled intravenous glucose tolerance test
GCP: Good Clinical Practice
GLUT: glucose transporter
HbA_1c_: hemoglobin A_1c_
HDL: high-density lipoprotein
HREC: Human Research Ethics Committee
HUVEC: human umbilical vein endothelial cells
LDL: low-density lipoprotein
MRI: magnetic resonance imaging
OPLS: Orthogonal Partial Least Squares
ORAC: oxygen radical capacity absorbance
OTUs: Operational Taxonomic Units
PARQ: physical activity readiness questionnaire
PCA: Principal Component Analysis
QC: quality control
ROS: reactive oxygen species
SA: South African
SAT: subcutaneous adipose tissue
SMC: Swedish Metabolomics Centre
SOD: superoxide dismutase
T2D: type 2 diabetes
TBARS: thiobarbituric acid reactive substance
VAT: visceral adipose tissue
VCAM: vascular cell adhesion molecule
