# Supplementary material for: An Exercise Intervention to Unravel the Mechanisms Underlying Insulin Resistance in a Cohort of Black South African Women: Protocol for a Randomized Controlled Trial and Baseline Characteristics of Participants
Source: JMIR Res Protoc. 2018 Apr 18;7(4):e75. doi: 10.2196/resprot.9098 (PMC5932332; doi:10.2196/resprot.9098)
Supplement: Multimedia Appendix 1 [file resprot_v7i4e75_app1.pdf]

Multimedia Appendix 1: Focus group discussion open-ended guide questions

Number	Question
1	Can you please briefly share with us what you have been up to since we last saw you?
2	What is the first thing that comes to mind when you hear the word ‘exercise’?
3	What are your perceptions about exercise?
4	What are some of the things that influenced you to attend the exercise sessions?, this can either be barriers or motivating factors.
5	What did you like the most the exercise sessions?
6	What did you not like much about the exercise sessions?
7	How did the exercise training affect your daily life?
8	What did your friends and family have to say when they found out that you are exercising?
9	Is there anything that could be changed about the exercise training sessions to encourage you to continue exercising and for your friends and family to start exercising?
10	Of all the questions that were discussed today, is there one which you feel was the most important to you?
	**Key Informant Interview Question**
1	How did you experience the exercise sessions?
