# Supplementary material for: An Exercise Intervention to Unravel the Mechanisms Underlying Insulin Resistance in a Cohort of Black South African Women: Protocol for a Randomized Controlled Trial and Baseline Characteristics of Participants
Source: JMIR Res Protoc. 2018 Apr 18;7(4):e75. doi: 10.2196/resprot.9098 (PMC5932332; doi:10.2196/resprot.9098)
Supplement: Multimedia Appendix 2 [file resprot_v7i4e75_app2.pdf]

Multimedia Appendix 2: Adipose tissue genes of interest

**GENES**	**ID**
LDL receptor related protein (LRP10)	Hs00204094_m1
Ribosomal protein lateral stalk subunit PO (RPLPO)	Hs99999902_m1
Peroxisome proliferator-activated receptor gamma (PPARγ)	Hs01115513_m1
Lipoprotein lipase (LPL)	Hs00173425_m1
Diacylglycerol O- acyltransferase 2 (DGAT2)	Hs01045913_m1
Adipose triglycerides lipase (ATGL)	Hs00386101_m1
Lipid droplet-associated protein (PLIN1)	Hs00160173_m1
Tumor necrosis factor alpha (TNFa)	Hs00174128_m1
Interleukin 10 (IL10)	Hs00961622_m1
Adiponectin (ADIP)	Hs00605917_m1
Leptin (LEP)	Hs00174877_m1
Macrophage migration inhibitory factor (MIF)	Hs00236988_g1
Nuclear factor kappa B (NF-kB)	Hs00765730_m1
Toll like feceptor 4 (TLR4)	Hs01060206_m1
Monocyte chemoattractant protein 1 (MCP1)	Hs00234140_m1
Catalase (CAT)	Hs00156308_m1
Superoxide dismutase (SOD)	Hs00533490_m1
Nitric oxide synthase 3 (NOS3)	Hs01574665_m1
Insulin receptor substrate 1 (IRS1)	Hs00178563_m1
Glucose transporter type 4 (GLUT4)	Hs00168966_m1
Serine/threonine-protein kinase 1 (SMG1)	Hs00979691_m1
